# Idiopathic Pneumatosis of Small Bowel and Bladder

**DOI:** 10.7759/cureus.8313

**Published:** 2020-05-27

**Authors:** Anupam K Gupta, Oscar A Vazquez, Miguel Lopez-Viego

**Affiliations:** 1 Surgery, Charles E. Schmidt College of Medicine, Florida Atlantic University, Boca Raton, USA; 2 Surgery, Bethesda Hospital, Boynton Beach, USA

**Keywords:** pci, ec, surgery, gi, urology, pneumatosis

## Abstract

An 81-year-old woman presented with multiple episodes of loose bowel movements. CT scan of the abdomen and pelvis revealed pneumatosis cystoides intestinalis and asymptomatic emphysematous cystitis. The patient had an extensive workup with no obvious identified pathology to explain diffuse pneumatosis of the small bowel and bladder. Her symptoms improved with symptomatic management, empirical antibiotics, and no surgical intervention.

## Introduction

Pneumatosis of the intestine, otherwise known as pneumatosis cystoides intestinalis (PCI), is a rare radiological finding affecting about 0.03% of the population with causes ranging from benign to life-threatening [1,2]. There is no clear etiology for the pneumatosis, although it is hypothesized that it could be secondary to mechanical, infectious, or pulmonary causes, such as chronic obstructive pulmonary disease (COPD), leading to accumulation of bowel gases within the walls of visceral organs [3]. While infectious causes usually need emergent intervention, pulmonary causes can be managed conservatively; however, there is no clear consensus on when surgery is indicated besides when there is a risk of ischemia or bowel perforation [4]. One study found that up to 27% of patients presenting with benign pneumatosis underwent avoidable surgery [5]. Emphysematous cystitis (EC) is a potentially life-threatening condition that is characterized by air within the wall of the bladder as a result of infection by gas-forming organisms, but it may not always be the case. In cases where there is no clear etiology, medical management may be the most appropriate, as the pathogenesis may be similar to PCI [6-8]. However, it is unusual to see pneumatosis of the small bowel and bladder simultaneously.

## Case presentation

**Figure 1:**
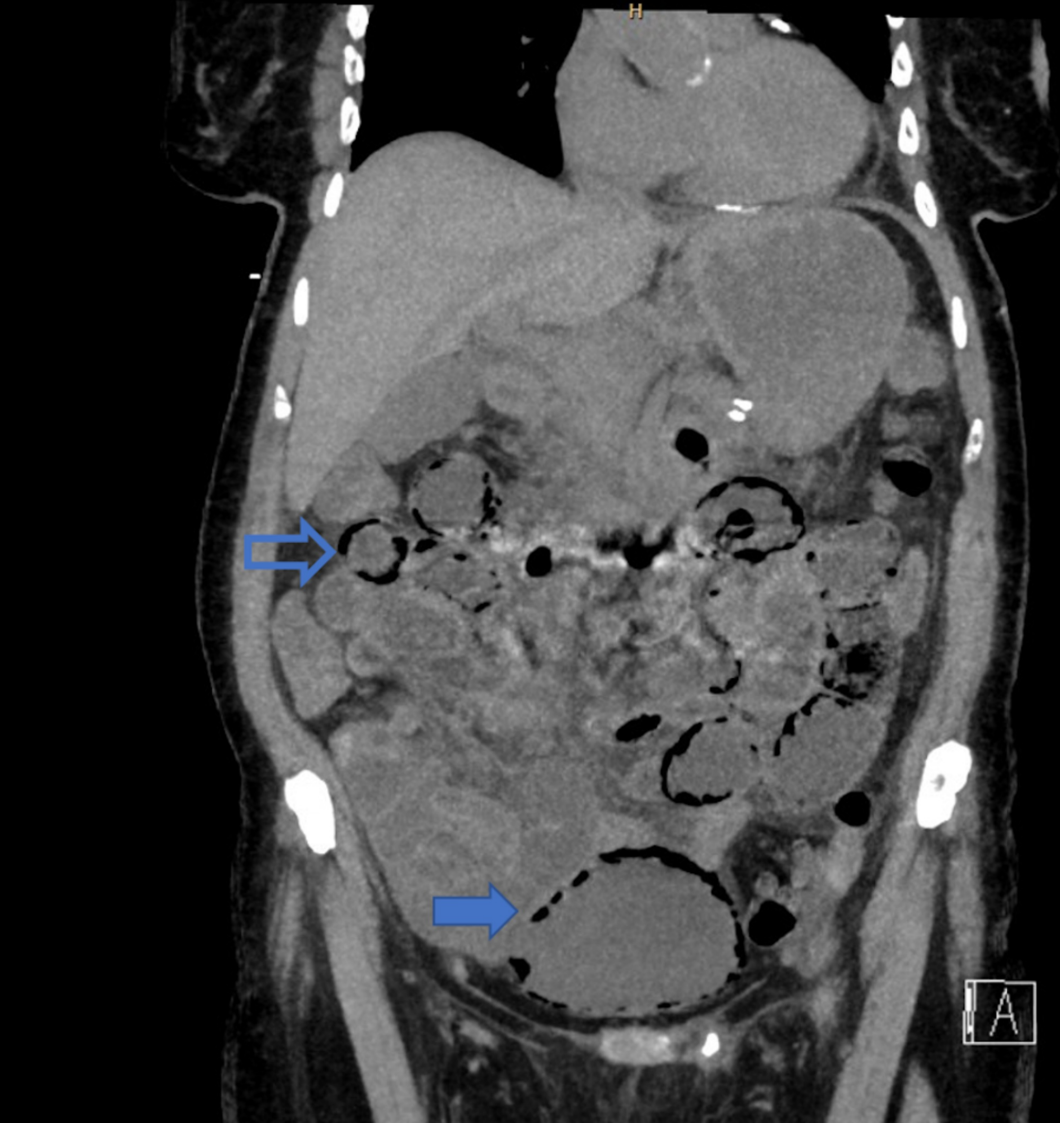
. Hollow arrow showing pneumatosis cystoides intestinalis (top) and solid arrow showing emphysematous cystitis (bottom).

**Figure 2:**
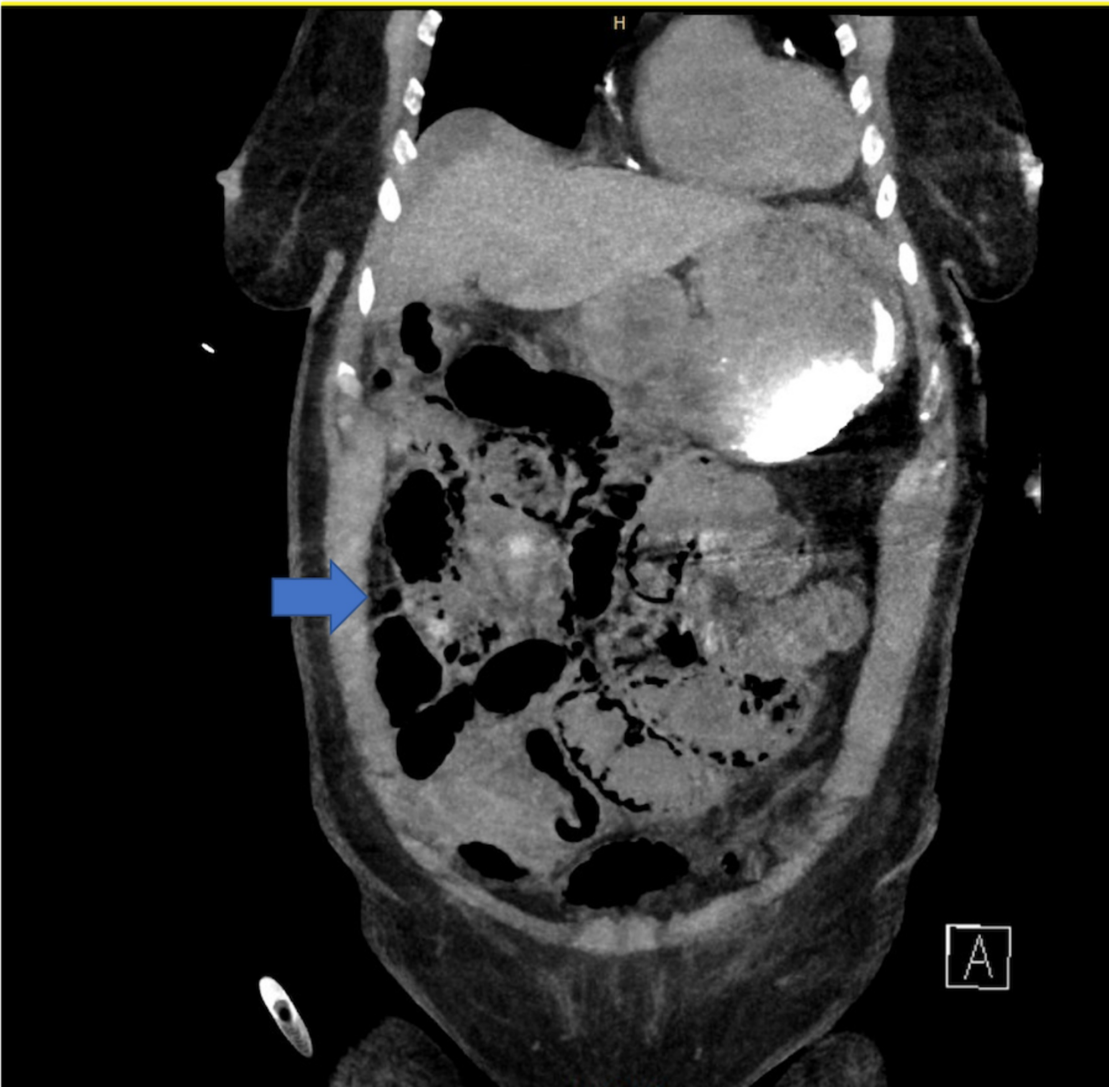
Diffuse pneumatosis of small bowel.

An 81-year-old woman with a medical history of hypertension, hyperlipidemia, and hypothyroidism presented to the emergency room for evaluation of diarrhea for two months’ duration, weakness, and fatigue. These multiple loose bowel movements were causing incontinence without hematochezia, melena, or abdominal pain. She denied associated symptoms, such as fever, symptom onset with a particular diet, or urinary symptoms. The patient had been evaluated the week prior for similar symptoms with an esophagogastroduodenoscopy (EGD), which showed evidence of moderately severe esophagitis with no bleeding in the distal esophagus, normal stomach, and normal duodenum. A colonoscopy performed the same day as the EGD was positive for large-mouthed diverticula found in the left colon, but the physical examination results were otherwise normal. Biopsies taken at that time from the esophagus, duodenum, and right and left colon revealed non-specific inflammatory changes on her pathology report. On physical examination, the patient had stable vital signs, was weak, was mildly distressed due to her symptoms, and had mild abdominal bloating. A complete blood count was unremarkable. A basic metabolic profile revealed a potassium level of 3.1 mmol/L, chloride 92 mmol/L, blood urea nitrogen of 27 mg/dL, and a creatinine of 1.5 mg/dL from a baseline of 1.0 mg/dL. Liver function tests and lipid profile were within normal limits. The patient had a mild elevation of troponins to a maximum of 0.21, which immediately trended down on subsequent follow-up troponin tests. An electrocardiogram and echocardiogram did not reveal any abnormalities. Further workup included a CT scan of the abdomen and pelvis with intravenous contrast, which revealed diffuse pneumatosis of the small bowel (Figure 1) and bladder (Figure 2).

**Figure 3:**
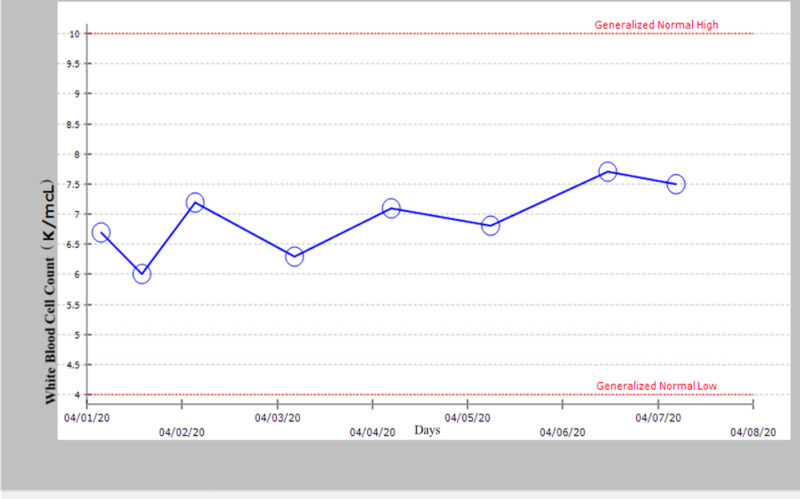
White blood count within normal limits during hospital stay.

The patient was immediately initiated on empirical antibiotic treatment with piperacillin-tazobactam and was evaluated for a possible infectious source. This included urinalysis, stool analysis, and cultures of the urine, blood, and stool. Studies were positive for *Klebsiella pneumoniae* growth in the urine culture subsequently treated with a seven-day course of piperacillin-tazobactam. The patient had a Foley catheter placed to decompress the bladder with no evidence of hematuria. In light of the diffuse pneumatosis, the patient was placed on bowel rest for two days and started on a diet after that. During her entire course of seven days at the hospital, the patient was afebrile, had good urine output, and had no abdominal symptoms other than loose stools, which improved with colestipol, a bile acid sequestrant. Blood work during her stay was unremarkable except for an elevation in creatinine, which resolved with hydration, and the patient was discharged on day 7 on a regular diet (Figure 3).

## Discussion

The exact etiology of PCI is unknown; however, it is noted that it may be related to increased intraluminal pressure caused by ileal surgery and colonoscopies, as well as other causes such as COPD, connective tissue disorders, and ingestion of sorbitol or lactulose [9]. As mentioned previously, it is theorized that the causes of PCI may be infectious, pulmonary, or mechanical. The infectious theory of PCI is based on fermenting Clostridia and Escherichia coli localizing to the submucosa and leading to the production of hydrogen gas, which is retained by the submucosa and lymphatic channels [10]. This theory is further supported by the observed resolution of pneumatosis with the use of metronidazole [11]. Pulmonary causes of PCI are theorized to be from gas freed by ruptured alveoli, which travels through the mediastinum into the perivascular spaces in the intestinal wall [12]. Mechanically speaking, it is theorized that bowel gas is pushed through a mucosal defect into lymphatic channels and spread distally by peristalsis; yet, this phenomenon does not explain hydrogen in the cysts formed [13,14].

Patients with PCI do not have a characteristic presentation. They may be asymptomatic or report concerns of pain with abdominal distension, diarrhea, and hematochezia, with a mortality rate that may reach up to 75% [15]. Furthermore, patients with EC present with classic lower urinary tract infection (UTI) signs, such as urgency and dysuria, along with possible pneumaturia. It is theorized to be caused by gas-producing organisms, high glucose concentration in tissues, and impaired tissue perfusion, as is often seen in middle-aged women with diabetes. Other risk factors for EC are chronic UTIs, indwelling urethral catheters, urinary tract outlet obstruction, or neurogenic bladders [6,7].

Laboratory tests that may be helpful include white blood cell count, aspartate aminotransferases, alanine aminotransferases, alkaline phosphatase, pH, bicarbonate, lactic acid, and amylase. These tests must be coupled with physical exam findings such as pain, diarrhea, fever, tenderness, rectal blood loss, and hypotension, as these can indicate increasing severity of presentation, which may require surgery [15]. For PCI, CT is more sensitive than plain radiography in distinguishing it from intraluminal air or submucosal fat since it can visualize the presence of air in the bowel wall. Additionally, CT is able to detect other more worrisome findings that may suggest an underlying cause of PCI (bowel wall thickening, altered contrast mucosal enhancement, dilated bowel, soft tissue stranding, portal air, and ascites) [16]. EC is also better diagnosed by CT, as it can better visualize the gas in the bladder wall. CT can also differentiate EC from vesicocolic fistula, intra-abdominal abscess, or an adjacent neoplastic malignancy [17].

Oxygen therapy has been suggested as the primary treatment for asymptomatic PCI, with antibiotic treatment reserved for EC, as it is most commonly due to bacterial causes if there are signs of infection. Otherwise, it is treated as PCI, even with the presence of free intraperitoneal air [18,19]. Oxygen treatment is based on increasing the partial pressure of oxygen in the blood and thus increasing the pressure gradient of the gas in the cysts. The cysts then release gases contained within them and refill with oxygen, which is then metabolized, leading to resolution [9,19]. Surgery is mostly indicated for intestinal and extra-intestinal complications from PCI and EC, such as obstruction caused by cysts, perforation from stercoral ulceration, adhesions, or compression of adjacent structures by large masses of cysts [20]. The presenting patient had diarrhea, mild abdominal distention, and no urinary symptoms concerning for UTI. It was only on CT scan that there was a manifestation of both PCI and EC. Laboratory results revealed an elevated troponin level and an elevated creatinine level, which resolved with fluid hydration. She had a positive history for colonoscopy and EGD a week before for her diarrhea workup, which could cause PCI, but it would not explain EC. It may be possible that it was a bacterial manifestation causing EC as there was an infectious source based on the positive urine culture; however, with blood cultures and repeat urine cultures being negative, this was unlikely the cause of the pneumatosis. The patient was ultimately discharged after completing seven days with a broad-spectrum antibiotic treatment for the UTI.

## Conclusions

Idiopathic, simultaneous asymptomatic pneumatosis of the small bowel and bladder is unusual and can be managed conservatively in a non-operative fashion if there are no concerns for intra- or extra-intestinal complications associated with PCI and/or EC.
